# Supplementary material for: Iterative improvement in the automatic modular design of robot swarms
Source: PeerJ Comput Sci. 2020 Dec 7;6:e322. doi: 10.7717/peerj-cs.322 (PMC7924708; doi:10.7717/peerj-cs.322)
Supplement: Supplemental Information 3 [file peerj-cs-06-322-s003.zip › argos3/doc/api/standalone/a00421_source.html]

ARGoS: plugins/robots/eye-bot/control\_interface/ci\_eyebot\_light\_sensor.h Source File


- Main Page
- Related Pages
- Namespaces
- Classes
- Files

- File List
- File Members

# plugins/robots/eye-bot/control\_interface/ci\_eyebot\_light\_sensor.h

Go to the documentation of this file.

```
00001 
00032 #ifndef CCI_EYEBOT_LIGHT_SENSOR_H
00033 #define CCI_EYEBOT_LIGHT_SENSOR_H
00034 
00035 namespace argos {
00036    class CCI_EyeBotLightSensor;
00037 }
00038 
00039 #include <argos3/core/control_interface/ci_sensor.h>
00040 #include <argos3/core/utility/math/angles.h>
00041 #include <vector>
00042 
00043 namespace argos {
00044 
00045    class CCI_EyeBotLightSensor : public CCI_Sensor {
00046 
00047    public:
00048 
00053       struct SReading {
00054          Real Value;
00055          CRadians Angle;
00056 
00057          SReading() :
00058             Value(0.0f) {}
00059 
00060          SReading(Real f_value,
00061                   const CRadians& c_angle) :
00062             Value(f_value),
00063             Angle(c_angle) {}
00064       };
00065 
00066       typedef std::vector<SReading> TReadings;
00067 
00068    public:
00069 
00070       CCI_EyeBotLightSensor();
00071       virtual ~CCI_EyeBotLightSensor() {}
00072 
00076       const TReadings& GetReadings() const;
00077 
00078 #ifdef ARGOS_WITH_LUA
00079       virtual void CreateLuaState(lua_State* pt_lua_state);
00080 
00081       virtual void ReadingsToLuaState(lua_State* pt_lua_state);
00082 #endif
00083 
00084    protected:
00085 
00086       TReadings m_tReadings;
00087    };
00088 
00089    std::ostream& operator<<(std::ostream& c_os, const CCI_EyeBotLightSensor::SReading& s_reading);
00090    std::ostream& operator<<(std::ostream& c_os, const CCI_EyeBotLightSensor::TReadings& t_readings);
00091 
00092 }
00093 
00094 #endif
```

---

Generated on 10 Jul 2018 for ARGoS by 
 1.6.1
